# Machine Learning Refutes Loss of Smell as a Risk Indicator of Diabetes Mellitus

**DOI:** 10.3390/jcm10214971

**Published:** 2021-10-26

**Authors:** Jörn Lötsch, Antje Hähner, Peter E. H. Schwarz, Sergey Tselmin, Thomas Hummel

**Affiliations:** 1 Institute of Clinical Pharmacology, Goethe University, Theodor Stern Kai 7, 60590 Frankfurt am Main, Germany; 2 Fraunhofer Institute for Translational Medicine and Pharmacology ITMP, Theodor-Stern-Kai 7, 60596 Frankfurt am Main, Germany; 3 Smell & Taste Clinic, Department of Otorhinolaryngology, Technische Universität Dresden, Fetscherstrasse 74, 01307 Dresden, Germany; Antje.haehner@ukdd.de (A.H.); thomas.hummel@tu-dresden.de (T.H.); 4 Department of Internal Medicine III, Technische Universität Dresden, Fetscherstrasse 74, 01307 Dresden, Germany; Peter.schwarz@ukdd.de (P.E.H.S.); sergey.tselmin@ukdd.de (S.T.)

**Keywords:** human olfaction, diabetes mellitus, machine-learning, data science, patients

## Abstract

Because it is associated with central nervous changes, and olfactory dysfunction has been reported with increased prevalence among persons with diabetes, this study addressed the question of whether the risk of developing diabetes in the next 10 years is reflected in olfactory symptoms. In a cross-sectional study, in 164 individuals seeking medical consulting for possible diabetes, olfactory function was evaluated using a standardized clinical test assessing olfactory threshold, odor discrimination, and odor identification. Metabolomics parameters were assessed via blood concentrations. The individual diabetes risk was quantified according to the validated German version of the “FINDRISK” diabetes risk score. Machine learning algorithms trained with metabolomics patterns predicted low or high diabetes risk with a balanced accuracy of 63–75%. Similarly, olfactory subtest results predicted the olfactory dysfunction category with a balanced accuracy of 85–94%, occasionally reaching 100%. However, olfactory subtest results failed to improve the prediction of diabetes risk based on metabolomics data, and metabolomics data did not improve the prediction of the olfactory dysfunction category based on olfactory subtest results. Results of the present study suggest that olfactory function is not a useful predictor of diabetes.

## 1. Introduction

Functional changes in the sense of smell have been described as accompanying symptoms of an increasing number of disorders [1]. These include otorhinolaryngologic disorders such as chronic rhinosinusitis, exposure to various environmental or occupational toxins [2], infectious diseases such as COVID19 [3], internal diseases like renal dysfunction [4], and neurological disorders such as Parkinson’s disease [5,6], Alzheimer’s disease [7], or multiple sclerosis [8]. For example, olfactory deficits are considered signs of developing central nervous complications of HIV infection [9], early signs of Parkinson’s disease [10,11,12], or signs of beginning cognitive dysfunction [13].

Olfactory dysfunction also occurs with higher prevalence in patients with diabetes [14,15,16,17,18,19,20]. It affects odor identification [14,21] as well as olfactory thresholds [22] and other olfactory functions [15]. Microvascular disease, renal dysfunction, or neuropathy have been attributed to it [19,23,24,25], among others. For example, the presence of macrovascular disease was found to be associated with olfactory dysfunction [17]. Olfactory dysfunction has been suggested to predict the development of dementia in elderly patients with type 2 diabetes [26]. Thus, while diabetes and olfactory symptoms were often found together, it remains unclear whether this is true for early stages before the manifestation of the disease, which would qualify olfactory tests as an early marker for the development of diabetes. In analogy to the increasing recognition of olfactory symptoms as early signs of several diseases characterized by loss of the sense of smell at later stages, including Parkinson’s disease [27] or multiple sclerosis [28], olfactory symptoms have been contemplated as early signs of diabetes or its aggravation toward cognitive decline [29].

Early diagnosis of diabetes is an active research topic [30,31] that has led to several solutions summarized in Table 1. Several markers have been proposed related to genetics, laboratory chemistry, physical activity, demographics, and others, which are used individually or in combination in diabetes risk scores [32] or as machine-learning based predictors [30].

Considering the utility of olfactory signs as early diagnostic markers for various diseases and the olfactory symptoms reported from patients with diabetes, the present study pursued the hypothesis that olfactory symptoms might already be present in the early stages of the disease and therefore add to the list of predictive markers for diabetes [44]. In the positive case of the present evaluations, loss of smell could be added as an early sign, either as a screening tool or, probably more realistically, as a signal detected during diagnostic procedures performed for smell problems, or diabetes could be added as another possible cause of such problems as a differential diagnosis. Indeed, early detection of diabetes mellitus type 2 is still often accidental; hence, connecting a further clinical sign with this potential diagnosis might increase the diabetes detection rate [30,31].

## 2. Materials and Methods

### 2.1. Study Design and Participants

The study was performed in accordance with the Declaration of Helsinki on Biomedical Studies Involving Human Subjects. It was approved by the ethics committee at the University Clinic of Dresden (approval number Ek328122008). All participants provided informed written consent. This was a cross-sectional study. The cohort included patients who reported to medical consulting at the diabetes outpatient clinic of the medical clinic III of the University Clinic Dresden. Measurements took place between 2009-02-17 Coordinated Universal Time (UTC) and 2010-03-24 UTC.

A total of 164 volunteers, i.e., 62 men and 102 women, aged 18–69 years (mean ± standard deviation: 52.8 ± 12.8 years) seeking medical consulting for possible diabetes participated. **Inclusion criteria** were age 18 years and older, non-smoking, absence of pregnancy, absence of neurodegenerative disorders such as Parkinson’s or Alzheimer’s disease, absence of other disorders that are strongly associated with olfactory loss, e.g., chronic rhinosinusitis. The sample size was chosen to exceed that of *n* = 111 used in a study on associations of diabetes with olfactory symptoms [17]. A formal case number calculation was not performed. The study included a selected sample of patients consecutively admitted to a specialty internal medicine unit for possible diabetes risk. This is certainly different from a random sample from the general population. With respect to olfactory function, potential confounders of olfactory function such as occupational exposure to toxic substances or the use of certain medications [45,46] were recorded and used to check the obtained results for major biases.

### 2.2. Data Acquisition

#### 2.2.1. Diabetes Risk Assessment

A diabetes risk score [32] was used, the validated German version of which is called “FINDRISK” [47]. The score is obtained from the multiple-choice responses to eight questions about (i) age, (ii) a family member with diabetes, (iii) the waist circumference (iv) physical activity, (v) consumption of vegetables, fruits, berries or dark bread (vi), antihypertensive medication, and (vii) knowledge about too high blood sugar levels and (viii) the body mass index. A FINDRISK score ≥ 9 has been found to predict drug-treated diabetes at a sensitivity of 0.78–0.81 and specificity of 0.76–0.77 in two different Scandinavian cohorts assessed in 1987 and 1992 [32]. The FINDRISK score is converted into five categories of diabetes risk in ascending order, i.e., “low risk” of 1% to develop diabetes in the next 10 years at scores < 7, “slightly increased” of 4% diabetes risk at scores [7, …, 11], “medium risk” of 17% diabetes risk at scores [12, …, 14], “high risk” of 33% diabetes risk at scores [15, …, 20] and “very high” risk of 50% to develop diabetes at scores > 20. The study participants completed the FINDRISK questionnaire at the beginning of the examinations, prior to olfactory testing.

#### 2.2.2. Clinical Testing of Olfactory Function

All participants underwent a standardized diagnostic procedure that included a detailed medical history and a detailed physical otorhinolaryngological examination [4,48]. In addition, olfactory function of all participants was assessed using an established clinical test (“Sniffin’ Sticks”, Burghart Instruments, Wedel, Germany) [49,50], which evaluated three sensory dimensions of odors comprising odor threshold (to phenylethylalcohol), odor discrimination (16 pairs of odors) and odor identification (16 odors). The olfactory functional category was obtained from the sum of scores for **T**hreshold, **D**iscrimination, and **I**dentification (TDI) subtests, with a range between 1 and 48 points and allows to categorize subjects as normosmic (>30.5), hyposmic (16.5–30.5), and functionally anosmic (<16.5), based on normative scores obtained in more than 9000 healthy subjects [51].

#### 2.2.3. Metabolomics Testing

Blood was withdrawn in all participants to assess the following parameters: triglycerides, cholesterol, low-density lipoprotein [LDL], high-density lipoprotein [HDL], glycohemoglobin [HbA1c], glucose, fatty acids, proinsulin, C-peptide, and insulin), and in addition, for glucose, free fatty acids, proinsulin, C-peptide, and insulin, the differences between the data measured 120 min after a glucose challenge to the respective individual baseline values. Concentration analyses were proofed in the local clinical routine laboratory. A diagnosis of diabetes was made using plasma glucose criteria, i.e., fasting plasma glucose or 2 h plasma glucose before and after a standard oral glucose tolerance test [52].

### 2.3. Data Analysis

**Figure 1:**
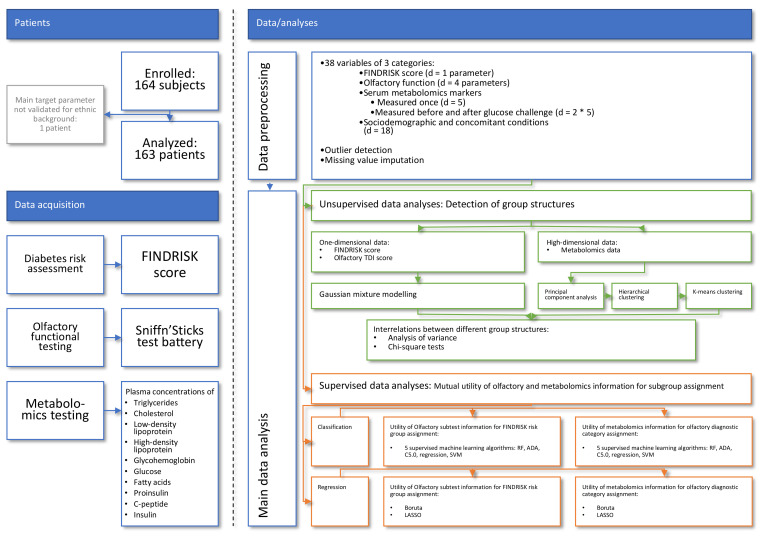
Flowchart showing the number of patients, the main items of data acquisition, and the steps of data analysis. The main steps of data analysis ranged from preprocessing to unsupervised and supervised analyses that assessed the extent to which olfactory and metabolomics databased subgroup assignments were mutually identified from the respective information. The figure has been created using Microsoft PowerPoint^®^ (Redmond, WA, USA) on Microsoft Windows 11 running in a virtual machine powered by VirtualBox 6.1 (Oracle Corporation, Austin, TX, USA).

The programming work for this report was performed in the R language [53] using the R software package [54] (version 4.0.5 for Linux), which is available free of charge in the Comprehensive R Archive Network (CRAN) at https://CRAN.R-project.org/. We performed all analyses on 1–12 cores of an Intel Core i9-7940X^®^ (Intel Corporation, Santa Clara, CA, USA) computer running Ubuntu Linux 20.04.2 LTS (Canonical, London, UK). An overview of the data analysis workflow is shown in Figure 1.

The dataset originally included *n* = 164 subjects and d = 38 variables, which included (i) diabetes risk as to the FINDRISK score and (ii–v) olfactory performance measures consisting of olfactory threshold, odor discrimination odor identification, and the TDI sum score. Metabolic blood serum markers included (vi) triglycerides, (vii) cholesterol, (viii) LDL, (ix) HDL, (x) Hba1c, and, measured before and after glucose challenge, (xi–xii) blood glucose, (xiii–xiv) free fatty acids, (xv–xvi) proinsulin, (xvii–xviii) C-peptide, and (xix–xx) insulin. Baseline demographic and medical parameters included (xxi–xxvi) subjects’ gender, age, body mass index (BMI), waist-to-hip ratio, and diastolic and systolic blood pressure. Other medical parameters included (xxvii–xxix) whether the subject smoked, had quit smoking and for how long, (xxx) alcohol consumption, and various possible risk factors for olfactory problems, including (xxxi) occupational exposure to volatile toxins, (xxxii–xxxvii) previous head trauma, craniofacial surgery, a history of sinusitis or nasal allergies, and concomitant diseases and medications. Finally, (xxxviii) the date of the examination was recorded.

The main data analysis was designed to detect associations between olfactory and metabolomics data and the risk of diabetes at different analytical levels. This included the identification of group structures in olfactory or metabolomics data and their mutual associations using classical statistical as well as machine learning-based methods. The analyses included unsupervised and supervised approaches. The idea behind the unsupervised analyses was that if the patterns found in the (i) metabolomics data match both the group structures in (ii) the odor data and the group structures in (iii) the diabetes risk, then the three lines of information are interdependent and relevance of the olfactory data to diabetes risk is supported by the data structure. This was implemented as Gaussian mixture modeling or principal component analysis (PCA [55,56]) and subsequent crosstabulation analyses. The idea behind the supervised analyses was that if a machine-learning algorithm can be trained with metabolomics information such that it is able to assign a patient to the correct diabetes risk group, and this can be improved if olfactory information is added or is reproduced if the metabolomics information is replaced with olfactory information, then it can be concluded that the olfactory information was relevant to the diabetes risk group structure of the data set.

#### 2.3.1. Data Prepossessing

To assess the need for data transformation, the distribution of continuous and ordinally scaled variables was examined by applying Kolmogorov–Smirnov tests [57] to the original data and after log, square root, or reciprocal data transformation. Necessary transformations for further analyses were performed in cases where this test was statistically significant. In the metabolomics data, outliers were detected following transformation by applying Grubbs tests [58]. Specifically, each outlier was replaced with a missing value. The procedure was iteratively repeated as long as significant results of Grubbs tests were obtained. A total of 12 outliers was detected and replaced with missing values. These calculations were performed using the R library “outliers” (https://cran.r-project.org/package=outliers [59]). Subsequently, values either originally missing or removed during outlier detection were imputed using k-nearest neighbors (kNN) with k = 3, calculated with the R-library “DMwR” (https://cran.r-project.org/package=DMwR [60]).

#### 2.3.2. Detection of Group Structures in Metabolomic and Olfactory Data

##### Assessment of Group Structures in One-Dimensional Olfactory and Diabetes Risk Data

Both the TDI olfactory score and the FINDRISK score of diabetic risk have predefined categories. That is, the TDI score translates to olfactory dysfunction categories of anosmia, hyposmia, and normosmia at the boundaries mentioned above with the description of the olfactory testing and the FINDRISK score is converted into categories of diabetes risk as described above with the description of the diabetic risk assessment. Both the TDI olfactory score and the FINDRISK scores were additionally examined for databased subgroups based on their probability density distributions described by Pareto density estimation (PDE) as a kernel density estimator that is particularly useful for detecting classes in one-dimensional data [61]. Modal structures were analyzed by fitting Gaussian mixture models (GMM) to the PDE as $p\left(x\right)=\sum_{i=0}^{M}w_{i}N\left(x|m_{i},s_{i}\right)$, where *N(x|m_i_,s_i_)* denotes Gaussian probability densities with expectation values *m_i_* and standard deviations *s_i_*. The *w_i_* denotes the mixture weights indicating the relative contribution of each of the M Gaussian components to the overall distribution. Models with M = [1, …, 5] Gaussian modes were tested, and the final model was selected based on the Bayesian Information Criterion (BIC [62]) and on likelihood ratio tests [63] comparing the goodness of fit between the GMM with the lowest value of BIC versus the corresponding simpler model, i.e., GMM with modes *M* versus GMM with modes *M–1*, on a Kolmogorov–Smirnov test comparing the distribution of the data predicted by the final model with the observed distribution, and on visual inspection of the quantile-quantile plots of the predicted versus observed data. The assignment of subjects to the identified subgroups was determined using Bayesian Theorem [62], which provides the decision limits for assigning a single observation to mode *M_i_* based on the calculation of posterior probabilities. An automated genetic algorithm was used for this purpose as implemented in our R library “DistributionOptimization” (https://cran.r-project.org/package=DistributionOptimization [64]).

##### Assessment of Group Structures in High-Dimensional Metabolomics Data

For the high-dimensional metabolomics data, a group structure was explored following projection of the data onto a low-dimensional plane followed by cluster analysis. Specifically, a data matrix was created containing the metabolic parameters measured at baseline (triglycerides, cholesterol, low-density lipoprotein (LDL), high-density lipoprotein (HDL), glycohemoglobin (HbA1c), glucose, fatty acids proinsulin, C-peptide, and insulin) and in addition, for glucose, Fatty acids, proinsulin, C-peptide and insulin, the differences between the data measured 120 min after a glucose challenge to the respective individual baseline values.

This 15 × 163 sized data space (*n* = 163 because one subject was excluded; see results section) was projected onto a two-dimensional space using PCA on scaled and centered data as the default settings of the R-library “FactoMineR“ (https://cran.r-project.org/package=FactoMineR [65]). Of the resulting main PCs, those with eigenvalues >1 were retained for clustering [66,67].

Clustering was subsequently performed according to the workflow proposed in [68]. That is, cluster analysis was carried out on the PCs and implemented as hierarchical clustering using Ward’s method [69] and the Euclidean distance. The clusters were subsequently consolidated using k-means clustering [70] to improve the initial partition obtained from hierarchical clustering [68]. These calculations were performed using the R libraries “FactoMineR“ (https://cran.r-project.org/package=FactoMineR [65]) and “cluster” (https://cran.r-project.org/package=cluster [71]). The cluster number was determined by calculating a total of 26 different indices available for this purpose using the R library “NbClust” (https://cran.r-project.org/package=NbClust [72]). The final number of clusters was determined by following the majority rule, i.e., the cluster count proposed by the largest number of indices. Cluster quality was evaluated by calculating the average Silhouette width [73]. The calculations were performed using the R libraries “cluster” and “fossil” (https://cran.r-project.org/package=fossil [74]).

#### 2.3.3. Investigation of Interrelations between Different Group Structures

##### Statistical Analysis of the Association between Odor Information and Diabetes Risk

Differences in FINDRISK groups were assessed for differences in olfactory test results using analysis of variance for repeated measures (rm-ANOVA), with the within-subject factor “olfactory subtest” (three levels, i.e., olfactory threshold. Odor discrimination, and odor identification) and the between-subjects factor “FINDRSIK score” (five levels comprising the five risk groups defined for this score). The α-level was set to 0.05 and corrected for multiple testing according to Bonferroni’s suggestion [75] when appropriate.

Furthermore, the groups of FINDRISK and olfactory scores, either predefined or found via Gaussian mixture modeling, were tested for significant associations which each other using χ^2^ tests [76]. Correlations between olfactory test results and metabolomic as well as demographic parameters were quantified by calculating Pearson’s r [76]. The correlation analyses were included as control items age since it is contributing to the FINDRISK score and is known to be correlated with odor identification performance [77] and the body mass index, which is also queried to calculate the FINDRISK score.

##### Evaluation of the Utility of Olfactory and Metabolomic Information in Predicting Diabetes Risk

The usefulness of olfactory and metabolomics information in predicting diabetes risk was further investigated using a supervised machine learning approach. **First**, machine-learned classifiers were trained with metabolomic, olfactory, or both types of information to assign an individual to the correct FINDRISK score-based diabetes risk group. Comparison of classification performance with different information types allowed assessment of the importance of olfactory information in predicting diabetes risk. **Second**, in addition to the classification approach, a machine learning-assisted regression approach was used. Specifically, feature selection [78,79] was performed to identify variables among the metabolomics and odor information that were relevant to the regression of either the FINDRISK score or the odor TDI score.

Training of the machine-learned of the **classifiers** was performed in 1000-fold repeated random subsampling cross-validation experiments as advised, for example, in [80], using Monte-Carlo [81] resampling to split the data set class-proportionally into two disjoint subsets, of which two-thirds of the original data served as the training data subset, and the remaining third served as the test data subset. This was done using the R library “sampling” (https://cran.r-project.org/package=sampling [82]). A negative control condition was implemented by repeating the training of the classifiers with permuted data, with the expectation that the classification would then be no better than guessing; otherwise, overfitting could not be ruled out. The classification was attempted for the original five categories of diabetic risk as defined for the FINDRISK score, and using only two classes of either lower or higher diabetic risk obtained by a combination of classes at possible breakpoints along with the ascending FINDRISK classes (e.g., class 1 versus 2–5, 1–2, versus 3–5, 1–3 versus 4–5, or 1–4 versus 5), or obtained by splitting the data at the breakpoint of FINDRISK = 9, as this was indicted in the 1992 report on the predictive performance of this score [32]. In addition, the clusters found in the unsupervised analyses were used as classification targets to account for any possible scenario of interactions between odor and metabolomics or diabetes data. The classification accuracy was primarily assessed as balanced accuracy [83], which is the mean of the predictive sensitivity and specificity for each olfactory dysfunction category and reflects the average of the proportion of correct results for each class. Other secondary measures of average classification performance across the olfactory dysfunction category included test sensitivity and specificity and negative and positive predictive values calculated using standard equations [84,85]. In addition, classification performance was assessed by calculating the area under the ROC curve (AUC-ROC) and the F1 measure [86,87]. These calculations were performed with the R libraries “caret” (https://cran.r-project.org/package=caret [88]) and “pROC” (https://cran.r-project.org/package=pROC [89]). The 95% confidence intervals (CI) of the classification performance parameters were determined as the range between 2.5% and 97.5% of the respective values during the 1000 cross-validation runs.

The classifiers were chosen to cover a variety of types so that the results do not depend on a single type of classifier. This included random forests [90,91] as a tree-based ensemble learner, adaptive boosting [92] as a tree-based classifier, a C5.0 non-hierarchical rule-based classifier [93], classical logistic regression [94] since this is often used in statistical analyses of similar data sets, and support vector machines (SVM [95]). The R libraries used for these calculations comprised “caret”, “xgboost” (https://cran.r-project.org/package=xgboost [96]), “C50” (https://CRAN.R-project.org/package=C50 [97], and “nnet” (https://cran.r-project.org/package=nnet [98]). Hyperparameters were set according to grid searches performed in preliminary assessments or during the training using the control function implanted in the “caret” library. For example, in the boosting, maximum tree depth = 5, eta = 0.25, and number of parallel trees = 5, or in random forests, 500 trees with $\left[0.5,1,1.5,2\right]\cdot \sqrt{n_{features}}$ and a maximum of seven nodes per tree were used. The large tree count was considered unproblematic as it has been shown that many trees do not confer a risk of increasing errors [99]. The C5.0 rule generating algorithm was used with the default hyperparameter settings of the C5.0 R library. In the case of negative results from the above analyses, similar machine learning analyses were used to assess whether the assignment of subjects to olfactory-parameter-based classes, either clinical categories or GMM-based subgroups, could be predicted with the metabolomics information. This was done to comprehensively test the possibility of an association between the present diabetes-related metabolomics and olfaction. The results are reported as a summary only, without all the details reported for the main analyses above.

In a second machine learning-based approach, the classification problem was retransferred into a regression problem, and variables relevant for linear regression of the diabetes risk or the olfactory performance were chosen using three different standard techniques of feature selection [78,79]. Feature selection was implemented using the “Boruta” approach, which is based on the random-forests algorithm. It provides a clear decision on whether a variable is important or not, derived from an internal 100-fold cross-validation approach and a statistical evaluation with *p*-values that are 0.01 by default [100]. These calculations were performed using the R package “Boruta” (https://cran.r-project.org/package=Boruta [100]) with default hyperparameter settings. In addition, Least Absolute Shrinkage and Selection Operators (LASSO [101]) were used for feature selection as implemented in the R library “glmnet” (https://cran.r-project.org/package=glmnet [102]), and another feature selection method was the analysis of the relative importance of variables for linear regression implemented in the corresponding R library “relaimpo” (https://cran.r-project.org/package=relaimpo [103]).

All feature selection methods were repeated 1000 times, with two-thirds of the data set being drawn from the original data set using Monte Carlo resampling. In each run, the features selected by the algorithms were collected. At the end of the 1000 runs for feature selection, the variables were categorized according to the frequency with which they appeared among the selected features. This was achieved using an item categorization technique implemented as computed ABC analysis [104] as a suitable method for feature selection in machine learning [105]. The algorithm divides each set of positive numbers, i.e., the absolute values of the protein loadings on the relevant PC, into three non-overlapping subsets named “A”, “B”, and “C” [106]. Subset “A” contains the “important few” which were retained. These calculations were performed using our R package “ABCanalysis” (http://cran.r-project.org/package=ABCanalysis [104]).

#### 2.3.4. Exploration of the Associations of Potential Confounders with Diabetes Risk

Associations of demographic factors, medications, concomitant diseases, or further risk factors for olfactory loss, such as sinonasal disease, nasal surgery, or professional exposition to toxins that could cause reduced olfactory function, with the FINDRISK score-based groups of diabetic risk were examined using standard statistical methods. For example, in view of reports that the loss of the sense of smell in patients with uncomplicated diabetes was modulated by hyperthyroidism or hypothyroidism [107,108], the distribution of these diseases and of additional factors across FINDRISK score-based groups of diabetic risk was analyzed by means of χ^2^ tests. The results of the crosstabulations were further examined in the case of significant χ^2^ tests by calculating the Pearson residuals to determine in which particular group the expected number of cases was significantly different from the observed number of cases. These calculations were done using the R library “vcd” (https://cran.r-project.org/package=vcd [109]). In addition, the effects of unequally distributed factors on the results of the olfactory subtests (threshold, discrimination, or identification; see above) were assessed by *t*-tests [110]. The α level was again set at 0.05 and corrected for multiple testing as described above. Finally, medications were screened by a pharmacological expert for known olfactory effects based on reported evidence [46,111].

## 3. Results

A 32-year-old woman of Asian background was excluded from the analysis because the FINDRISK diabetes risk score has so far only been validated for Caucasians. The analyzed *n* = 163 individuals were between 18 and 69 years old (mean value ± standard deviation: 52.9 ± 12.7 years). The olfactory test results and diabetes risk score data were complete. Olfactory functional categories in the cohort included one patient with anosmia, 39 subjects with hyposmia, and 123 subjects who were normosmic. The categories of the FINDRISK score predicting the risk of diabetes as low, slightly increased, medium, high, or very high were presented by 19, 68, 36, 33, and 7 subjects, respectively. By plasma glucose criteria, 94 participants were healthy, 64 were prediabetic, and 6 had manifest diabetes.

**Figure 2:**
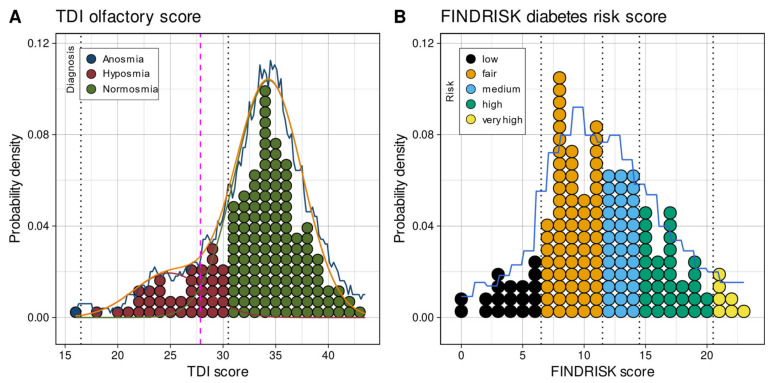
Distribution and group structures of one-dimensional main data: TDI olfactory score and FINDRISK score of diabetic risk. The dotplots show the individual values arranged in bins along with the range of values. The dots are colored according to predefined groups. Group boundaries are shown as vertical dashed black lines. The distribution of the data is shown as probability density function (PDF) estimated by means of the Pareto density estimation (PDE [61]; blue line) and overlaid on the histogram-like dotplots. For the olfactory TDI score, a GMM fit is shown as a red line, and the *M* = 2 single mixes are indicated as different colored lines. The Bayesian boundary between the Gaussians is indicated as a perpendicular magenta dashed line. The figure has been created using the R software package (version 4.0.5 for Linux; https://CRAN.R-project.org/ [54]) and the library “ggplot2” (https://cran.r-project.org/package=ggplot2 [112]). The colors were selected from the “colorblind_pal” and “stata_pal” palettes provided with the R library “ggthemes” (https://cran.r-project.org/package=ggthemes [113]).

**Figure 3:**
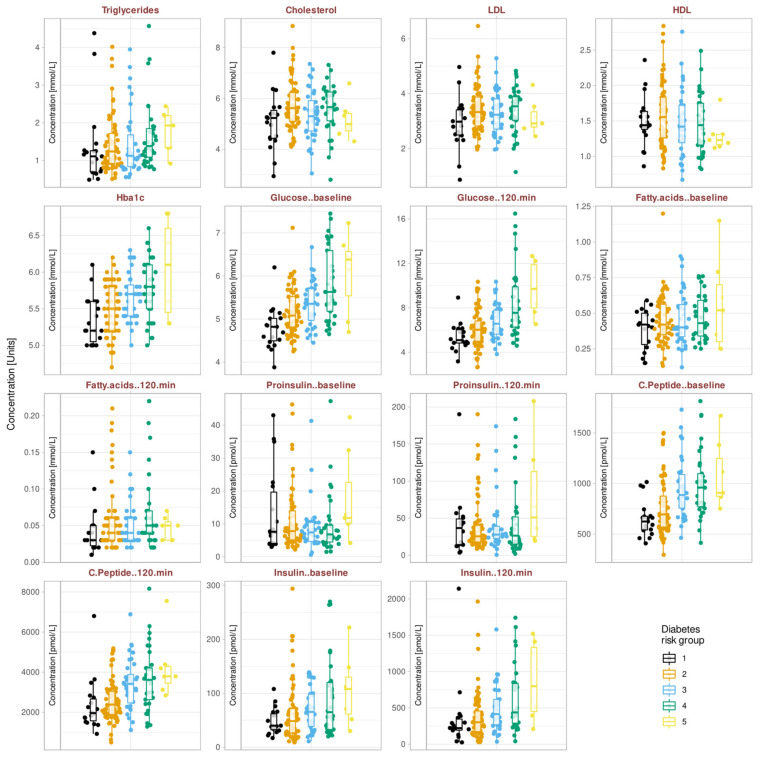
Raw metabolomics data. The data are plotted separately for the five diabetes risk groups according to the FINDRISK score (1 = “low risk”, 2 = “slightly increased risk”, 3 = “medium risk”, 4 = “high risk”, and 5 = “very high risk”). Individual data are shown as dots; six outliers removed from the further analysis are not shown to ensure discernibility of the projection of data points onto the ordinate. The original data are overlaid with boxplots, constructed using the minimum, quartiles, median (solid line within the box), and maximum. The whiskers add 1.5 times the interquartile range (IQR) to the seventy-fifth percentile or subtract 1.5 times the IQR from the twenty-fifth percentile. The figure has been created using the R software package (version 4.0.5 for Linux; https://CRAN.R-project.org/ [54]) and the R package “ggplot2” (https://cran.r-project.org/package=ggplot2 [112]). The colors were selected from the “colorblind_pal” palette provided with the R library “ggthemes” (https://cran.r-project.org/package=ggthemes [113]).

An overview of the distributions of TDI odor scores, FINDRISK score, and subject age is shown in Figure 2. Additional demographic and medical details of the cohort are summarized in Table 2. The metabolomics data (Figure 3) contained six missing values in which the values after the glucose challenge had not been measured. These data, and the 12 removed outliers detected based on significant Grubbs tests, were imputed.

### 3.1. One- and High-Dimensional Group Structures in Metabolomic and Olfactory Data

The one-dimensional FINDRISK diabetic risk scores were unimodally normally distributed (Figure 2). Unimodality was supported by the lowest BIC obtained with a Gaussian model with M = 1 modes as compared to models with M = [2,…, 5] modes, and by a non-significant Kolmogorov–Smirnov test at *p* = 0.282 obtained with for a single Gaussian in untransformed data. In the distribution of the olfactory TDI scores, bimodality was supported by the lowest BIC for M = 2 modes (BIC = 994.5056, 991.8270, 1014.8427, 1027.3479, and 1044.3476 for M = 1 to 5 modes, respectively) and a non-significant Kolmogorov-Smirnov test of *p* = 0.992 for the data predicted by a Gaussian mixture with M = 2 modes versus the observed data. Thus, two modes were supported with means = (24.9, 34.32), standard deviations = (3.42, 3.2) and weights (0.167, 0.83). The Bayesian decision boundary between the resulting groups of patients with different olfactory performances was calculated as TDI = 27.85 (Figure 2).

**Figure 4:**
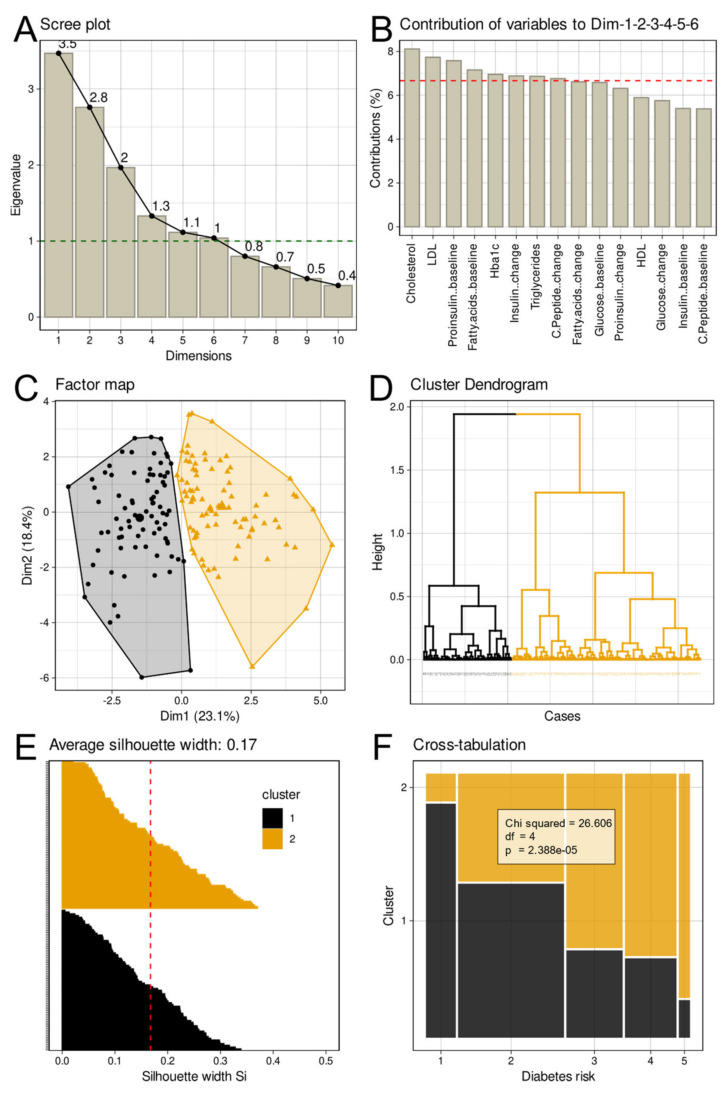
Results of principal component analysis (PCA) and Ward/k-means based clustering of the metabolomics data. (**A**): Scree-plot of the amount of variation of the data captured by each PC. The dashed horizontal reference dashed denotes the limit for PC selection for clustering set at an eigenvalue > 1 [66,67]. (**B**): Barplot of the contribution of each metabolomics parameter to PCs #1–#6 as the PCs with eigenvalues > 1. (**C**): Factorial plot of the individual data points on the principal component map, obtained following Ward clustering followed by k-means based cluster consolidation [68]. The colored areas visualize the cluster separation. (**D**): Cluster dendrogram obtained with the Ward algorithm. The two-cluster solution was the majority vote of 26 different indices [72] calculated to determine the number of clusters. (**E**): Silhouette plot [73] for the two-cluster solution. Positive values indicate that the sample is away from the neighboring cluster, while negative values would indicate that samples might have been assigned to the wrong cluster because they are closer to neighboring clusters than to their own cluster (not found). (**F**): Mosaic plot, visualizing the contingency table between the original group structure with respect to the diabetes risk (1 = “low risk”, 2 = “slightly increased risk”, 3 = “medium risk”, 4 = “high risk”, and 5 = “very high risk”) and the cluster identified on the PCA projection of the metabolomics data. The results of χ^2^ testing are indicated on the panel. The figure has been created using the R software package (version 4.0.5 for Linux; https://CRAN.R-project.org/ [54]) and the R packages “ggplot2” (https://cran.r-project.org/package=ggplot2 [112]) and “FactoMineR“ (https://cran.r-project.org/package=FactoMineR [65]). The colors were selected from the “colorblind_pal” palette provided with the R library “ggthemes” (https://cran.r-project.org/package=ggthemes [113]).

PCA projection of the high-dimensional metabolomics data (Figure 4) retained six PCs with eigenvalues > 1. Together, they explained 77.86% of the total variance. The variable’s contribution to these PCs was fairly similar, with a gradual decrease from total cholesterol to C-peptide (Figure 4). The metabolomics data contained a two-cluster structure (Figure 4). That is, a number of k = 2 clusters was the majority vote of the 26 different indices to determine the number of clusters. Hierarchical Ward clustering followed by consolidation using k-means clustering resulted in a two-cluster solution with *n* = 80 and 83 cases, respectively. Cluster quality was moderate with an average silhouette width of 0.17; only positive silhouette values were seen.

### 3.2. Interrelationships between Different Group Structures

#### 3.2.1. Results of Statistical Analyses of the Association between Olfactory Information and Diabetes Risk

For the FINDRISK score, the predefined clinical risk groups were examined for association with TDI-based groups of subjects with different olfactory performances. Cross-tabulation and subsequent χ^2^ statistics were negative for both variants of TDI-based groups, i.e., olfactory subgroups were not unequally represented among the five FINDRISK score-based diabetic risk groups (diabetic risk groups versus olfactory dysfunction categories: χ^2^ = 7.794, df = 8, *p* = 0.4538; diabetic risk groups versus GMM based groups found in the distribution of the TDI values: χ^2^ = 6.3792, df = 4, *p* = 0.1726). Negative results were also observed for the crosstabulations of olfactory parameter-based groups versus the two metabolomics-based clusters (olfactory dysfunction categories: χ^2^ = 2.2751, df = 2, *p* = 0.3206; SDI based groups: χ^2^ = 2.1043·10^−30^, df = 1, *p* = 1). In contrast, the five FINDRISK score-based groups were unequally represented among the two clusters found in the metabolomics data, in decreasing order of risk between clusters #1 and #2 (Figure 4). This was highly statistically significant with χ^2^ = 26.606, df = 4, *p* = 2.388 × 10^−5^.

Furthermore, the five groups of subjects with different diabetic risks did not differ in their performance in the olfactory tests of olfactory threshold, odor discrimination, and odor identification (rm-ANOVA: main effect of “FINDRISK score”: df = 4.158, F = 1.106, *p* = 0.356, interaction “FINDRSIK score” by “olfactory subtest”: df = 8.316, F = 0.728, *p* = 0.667). Only the expected result that the olfactory subtests differed statistically significantly among each other was obtained (main effect “olfactory subtest”: df = 2.316, F = 136.35, *p* < 2 × 10^−16^). Non-significant effects on olfactory subtest scores were also seen when the five FINDISK score-based groups were replaced by the two metabolomics-based clusters or by the two-class variant used in machine learning (see below), in which groups were combined at FINDRISK scores ≤ 11 versus FINDRISK score > 11 (i.e., combined FINDRISK classes 1 and 2 versus FINDRISK classes 3–5, ANOVA details not shown).

**Figure 5:**
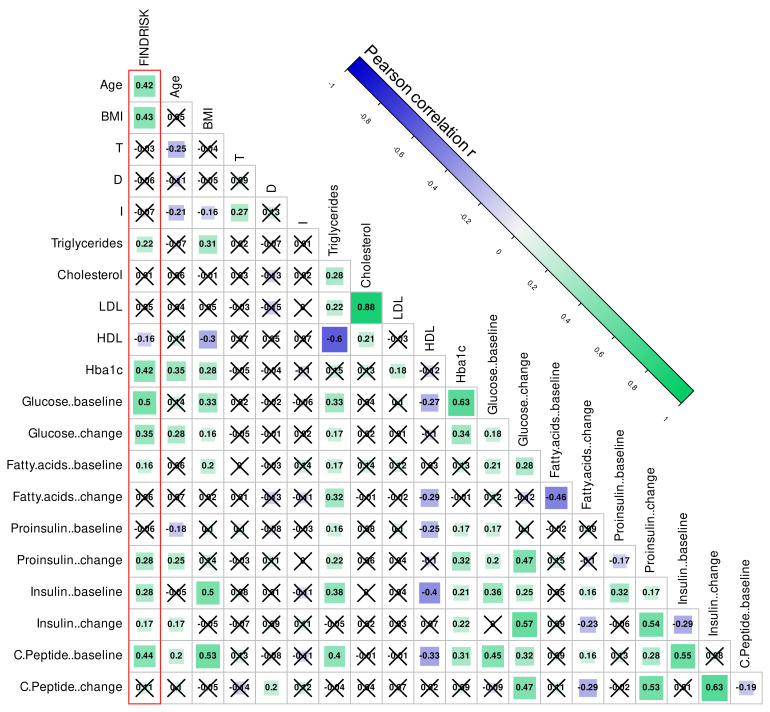
Correlations of metabolomics and olfactory data. Age and BMI are additionally included as control variables. In the lower-left part, the correlations are color-coded for both correlation strength and direction (bars in the upper right corner). The color-coding of the correlation ranges from the blue for a high negative correlation, to gray/white for no correlation, to green for a strong positive correlation. The more intense the color, the higher the correlation. The correlation strength is additionally coded by the size of the square symbolizing the correlation. Cell labels indicate Pearson’s r [114] values, crossed out if not statically significant. Correlations with the FINDRISK score as the main target of this analysis are marked with a red frame. The figure has been created using the R software package (version 4.0.5 for Linux; https://CRAN.R-project.org/ [54]) and the library “corrplot” (https://cran.r-project.org/package=corrplot [115]).

Correlation analyses underscored this lack of association of olfactory performance test scores with diabetes-related variables. Specifically, the correlation matrix (Figure 5) indicated significant correlations for the control items, i.e., age and body weight were correlated with the FINDRISK score, as they are both components of it, and age was correlated with the odor identification performance, which is a long-established relationship [77]. As expected, the FINDRISK score was also significantly positively correlated with most metabolomic parameters, except for a negative correlation with plasma HDL and some non-significant observations (Figure 5). However, none of the olfactory subtest scores was significantly correlated with any other element of the matrix except age.

#### 3.2.2. Utility of Olfactory and Metabolomic Information in Predicting Diabetes Risk

##### Machine-Learned Classification Approach

Machine-learned classifiers could be trained with the complete metabolomics information to better predict a subject’s assignment to the correct FINDRISK diabetes risk class than by guessing when creating a two-class problem with either low risk (combined FINDRISK classes 1 and 2) or higher risk (FINDRISK classes 3–5), i.e., FINDRISK scores ≤ 11 or FINDRISK score > 11, respectively. Using only the metabolomics information, the balanced classification accuracy and its 95% confidence interval obtained in the 1000 cross-validation runs were consistently above the 50% guess level for all included classifiers (Table 3). That this was not due to overfitting is evident from the results of training the algorithms with permuted metabolomics data, where the balanced classification accuracy was close to 50%, and its 95% CI always included the value of 50%. From these observations, it could be concluded that metabolomics data contained information relevant to the diabetes risk according to the FINDRISK score. For brevity, results with permuted data are shown only if the classifiers were successful with the original data (Table 3 and Table 4).

Contrasting observations were made with the odor data. That is, when the scores obtained in the subtests of olfactory threshold, odor discrimination, and odor identification were added to the list of features, the classification accuracy obtained with the metabolomics information alone did not improve. Furthermore, the olfactory information obtained in the three subtests did not provide a sufficient basis to train a classifier to assign a subject to the correct diabetes risk class better than by guessing, i.e., all 95% CIs of the classification accuracy included the value of 50% (Table 3).

Furthermore, when the classification target was changed from FINDRSIK-based classes to clinical olfactory categories (two classes consisting of either normosmia or reduced function, because the one anosmic patient had to be included in the same group as the hyposmic subjects), the metabolomics information did not provide enough information to allow correct group assignment of subjects, which was possible, as expected, when the olfactory subtest results were added to the list of features used to train the algorithms or when only the olfactory subtest results were used (Table 4). Again, using permuted features for training led to the failure of the classifiers to assign subjects to the correct olfactory category, which indicated that the successful classification was not an artifact of overfitting. These results further confirm that the current metabolomic parameters have no relation to olfaction. Finally, analogous assessments with slightly different classes such as the metabolomics clusters of the GMM based TDI olfactory classes provided similar results in terms of the mutual roles of metabolomics and olfactory information for the respective diabetes of olfactory classes (details not shown).

##### Machine-Learned Regression Approach

**Figure 6:**
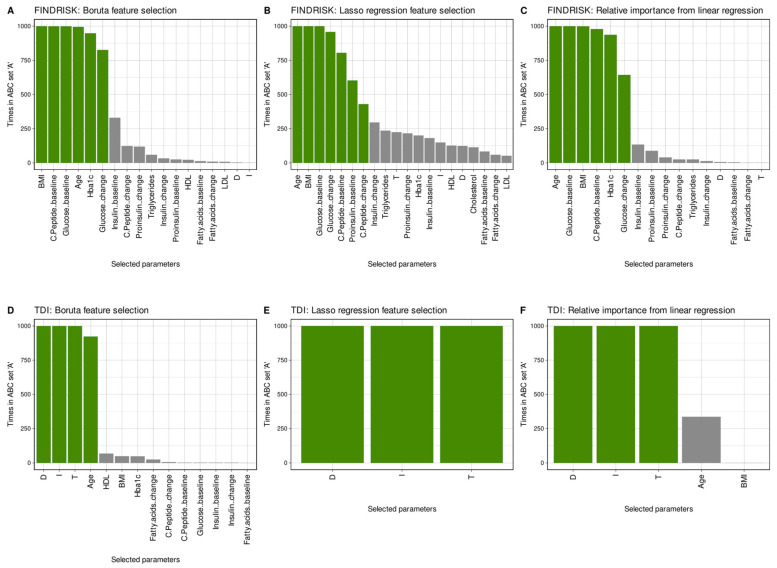
Relevant regressors of either diabetes risk or olfactory test score. Feature selection results for regression analyses using three different methods, including (i) the random forest-based “Boruta” method [100] (panels (**A**,**D**)), (ii) the least absolute shrinkage and selection operator (LASSO [101]) (panels (**B**,**E**)), and (iii) the analysis of the relative importance of variables for linear regression [103] (panels (**C**,**F**)), followed by the selection of the most relevant variables using the calculated ABC analysis [104,105]. The bar charts show the variables that were identified as important by the feature selection algorithms in 1000 runs on two-thirds of the instances selected by random Monte Carlo selection from the original dataset. The final feature set (green bars) indicates the members of the ABC set “A” that results from subjecting the number of selections as relevant variables in the 1000 replicates to item categorization via computerized ABC analysis. The size of the final feature set is the most common size of the set of selected features during the 1000 runs. Variable name abbreviations: T: olfactory threshold, D: odor discrimination, I: odor identification, BMI: body mass index, HDL: high-density lipoprotein (HDL cholesterol), LDL: low density lipoprotein. The figure has been created using the R software package (version 4.0.5 for Linux; https://CRAN.R-project.org/ [54]) and the R package “ggplot2” (https://cran.r-project.org/package=ggplot2 [112]).

Feature selection for regression of either diabetes risk quantified by the FINDRISK score or olfactory acuity quantified by the TDI score indicated similar features observed to be important in the classification tasks (Figure 6). Specifically, all feature selection methods used, including Boruta, LASSO, and the relative importance of variables for the linear regression method, revealed that metabolomic variables were relevant for the FINDRISK regression and olfactory variables were relevant for the TDI regression, with no crossover between variable groups. Age and BMI were important for the FINDRISK score, consistent with their contribution to that score, while age was also selected as a variable relevant to the regression analysis of the TDI score, but much less frequently than its components olfactory threshold, odor discrimination, and identification, but well consistent with the long-established age dependence of olfactory function [77].

### 3.3. Associations of Medical or Other Factors or Potential Confounders with Diabetes Risk

The diagnoses of prediabetes or diabetes had a significant effect on the assignment of subjects to the five FINDRISK-based diabetes risk groups (χ^2^ = 29.659, df = 8, *p* = 0.0002428), which was subsequently attributed to the overrepresentation, compared to a random group distribution, of subjects with manifest diabetes in the “high” diabetes risk group. However, prediabetes or manifest diabetes had no effect on subjects’ performance in the three olfactory diagnosis groups (analyses of variance: *p*-value always >0.7), and the representation of these subjects in the olfactory dysfunction category was random (χ^2^ 5.1975, df = 4, *p* = 0.2676).

The distribution of hypothyroidism was unequal across the FINDRISK-based groups (χ^2^ = 13.14, df = 4, *p* = 0.01061), owing to overrepresentation in the small group of very high risk of diabetes. However, none of the olfactory subtests were significantly affected by hypothyroidism or hyperthyroidism (unpaired *t*-tests of olfactory threshold, odor discrimination, odor identification versus presence or absence of comorbidity: uncorrected *p*-values always >0.05). All other comorbidities or prior surgeries were evenly distributed among the five diabetes risk groups, with the exception of postnasal drip (χ^2^ = 18.996, df = 4, *p* = 0.0007873), which was disproportionately more frequent in the medium diabetes risk group. Negative results were also seen in similar analyses of smoking behavior or professional exposure to substances that potentially affect the sense of smell. Gender was also equally distributed among the FINDRISK-based groups (χ^2^ = 1.791, df = 4, *p* = 0.7741).

Classes of medications taken by participants included antiarrhythmics, antiparkinsonian agents, cytostatics, antidiabetics, statins, fibrates, antihypertensives, analgesics, antiasthmatics, and antidepressants. Only the ingestion of antihypertensives was unequally represented among FINDRISK risk groups (χ^2^ = 29.271, df = 4, *p* = 6.885 × 10^−6^. However, the disparity did not correspond to increased risk, as would be expected from querying this mediation within the FINDRISK score, but was due to the underrepresentation of antihypertensives in FINDRISK class #2 and overrepresentation in class #3. None of the medications were unequally distributed among subjects with different olfactory dysfunction categories (χ^2^ tests: *p* always >0.45). Finally, manual screening identified no single drug with available reported evidence for effects on olfactory performance.

## 4. Discussion

The present analyses provided no support that functional information about a subject’s sense of smell captured in a standard clinical odor test that assesses three dimensions of the sense of smell, namely odor threshold, odor discrimination performance, and odor identification performance, is related in any way to metabolomics data commonly collected as part of diabetes diagnosis or to a validated score that quantifies an individual’s risk of developing diabetes within the next decade. While several lines of data analysis consistently succeeded in establishing an association of metabolomics data with the degree of diabetes risk, the same analyses failed to identify an analogous association for olfactory function. This was observed in a setting to predict diabetes risk, and most participants did not have diagnosed diabetes at the time of data collection. The results, therefore, discourage claims that the sense of smell could be used as a simple, non-invasive screening test for the early detection of diabetes.

The comprehensive data analysis was performed to corroborate the negative result regarding the usefulness of olfactory information obtained in a standard clinical test for the early prediction of diabetes against a random failure of the analysis. Therefore, the dataset was assessed using predefined clinical olfactory dysfunction categories or diabetes risk groups, and additional unsupervised subgroup detection was performed at multiple levels of analysis. All results showed that the analyses were consistently negative with respect to the above hypothesis, whereas they were consistently positive for the several included control studies whose results were known from the current state of olfactory or metabolomics science. Negative results of associations of olfactory information with metabolomics or diabetes risk were obtained using standard statistics implemented as analyses of variance or correlation analyses—association analyses of olfactory-based subject groups with metabolomics-based subject groups after data projection by PCA were also negative. Finally, five different supervised machine learning methods showed no utility of olfactory information to assign subjects to subgroups defined by metabolomics parameters or by the diabetic risk. Moreover, the reverse task of using metabolomic information to assign subjects to olfactory-defined subgroups after training machine learning algorithms also consistently failed. In contrast, the groups identified in the metabolomics data by the unsupervised analysis matched well with the given groups of diabetes risk, and the machine-learning algorithms were ell able to assign subjects to diabetic risk groups using the metabolomics information or to assign subjects to olfactory subgroups using the olfactory subtest information, but not vice versa.

The hypothesis of a benefit of olfactory information as an early sign of the development of diabetes, as the FINDRISK score is designed, was derived from suggestions in olfactory research, where more and more diseases have been associated with olfactory disorders in recent decades, and olfactory testing has been proposed as screening in some of these cases. Specifically, olfactory tests have been shown to predict neurological disorders such as Parkinson’s disease [10,11], Alzheimer’s disease [7,116], multiple sclerosis [8], dementia [117], or 5-year mortality in older adults [118]. For example, olfactory deficits are considered signs of developing central nervous complications of HIV infection [9], early signs of Parkinson’s disease [10], or signs of beginning cognitive dysfunction [13].

In diabetes, the relationship between the presence or progression of the disease and loss of olfaction is less clear. Proposed pathomechanisms include ideas about the prominent role of insulin in the olfactory system [23], with the olfactory bulb being the brain area with the highest rate of insulin transport, the highest level of insulin, and the highest level of insulin receptors [119,120,121], at least in rodents while a transcriptomics analysis of the human olfactory bulb did not identify insulin receptors but only insulin-like growth factor binding proteins 5 and 7 genes IGFBP5 and IGFBP7 [122]. Furthermore, in light of manifest diabetes, it has been hypothesized that polyneuropathic changes contribute to the loss of olfaction [19]. Olfactory dysfunction has been shown to be an early marker/predictor of cognitive dysfunction in type 2 diabetes. Based on this association, it may also be that the observed decline in olfactory function in diabetes is related to the onset of cognitive decline. The present findings also argue against the notion that changes in olfactory processing observed in early diabetes are related to diabetes itself. Rather, they suggest that changes in olfactory brain networks are indicative of changes in cognitive processing associated with diabetes [29]. In this regard, the present findings are consistent with the occasionally observed lack of olfactory involvement in the symptoms of diabetes [123], as well as a lack of correlation between olfactory function and duration of diabetes [124].

The present analyses targeted the FINDRISK score of diabetes risk and not diabetes. That is, except for six subjects, it is not known whether participants developed diabetes after the present analyses. The conclusions of a lack of association of olfactory dysfunction with later diabetes rely on the accuracy with which the FINDRISK score can detect later diabetes. However, the score was a useful tool for this cross-sectional study; a definitive association of early olfactory symptoms with later diabetes would have required a longitudinal study design. On the other hand, the present results are clearly and consistently negative, with no sign of at least a tendency for odor symptoms to be associated with diabetic risk or metabolomic changes. The prediction of the FINDRISK score also makes the present results not comparable to a recent proposal of a combined score based on support vector machines that performed best among a variety of algorithms, with a reported accuracy of 96% for diabetes detection [30]. The present lower balanced accuracies of the trained classifiers refer to the FINDRISK-related groups created using Gaussian mixture modeling. However, to definitively reject the usefulness of olfactory symptoms as an early sign of diabetes, this should be studied prospectively, with participants followed up long enough to detect diabetes or, if diabetes is diagnosed, so many years have passed that no reasonable association can be made anymore with the earlier recorded olfactory symptoms.

Cross-validation based on Monte Carlo resampling of two-thirds of the cases as the training dataset and applying the trained classifiers to the remaining one-third of the cases that formed the test dataset was performed to assess whether the metabolism information was useful to determine (i) diabetes risk and (ii) olfactory diagnosis. Conversely, a similar approach was used to assess if the olfactory information was useful to determine (i) olfactory diagnosis and (ii) diabetes risk. The machine learning-based classifiers were used here for knowledge discovery, not to create a clinical diagnostic tool as a common use of such algorithms. The latter might have required more complex cross-validation techniques, such as nested cross-validation [125] or separating a validation sample from the dataset exactly once at the beginning of the data analysis and using it only when the final classifiers are to be validated, or an independent sample could have been obtained in another study. All of these techniques are even more conservative than the training/testing splits used currently, and thus it is unlikely that the main conclusions, namely that metabolomics information is not useful for predicting olfactory diagnoses and olfactory information is not useful for predicting diabetes risk, would have been different with these approaches. This would imply the expectation that the more difficult the task was for the classifier, the better the classification accuracy, which is unlikely.

## 5. Conclusions

The results of the present study suggest that olfactory function is not a useful predictor of diabetes. The negative results clearly indicate that the classic diagnostic tools based on metabolic blood parameters are sufficient and that the sense of smell does not provide relevant additional information about early signs of diabetes. In addition, the lack of an association with impending diabetes suggests a reevaluation of the association with the consequences of diabetes [26], i.e., whether, for example, olfactory dysfunction predicts dementia in diabetics because there is a close association between the two, or whether olfactory dysfunction may be an early sign of the development of dementia but without a specific association with diabetes. Nevertheless, a definitive rejection of the sense of smell as a symptom for diabetes risk may require a prospective study directly involving later diabetes rather than the present FINDRISK score; however, the present results do not suggest to expect substantial positive results from such a study.

## Figures and Tables

**Table 1:** Summary of existing solutions for early diagnosis of diabetes mellitus type 2.

Early Sign	Method of Detection	Key Reference
Genetic variation	Genetic variation	[33]
Family history	Relatives with diabetes	[34]
Age, family history, waist circumference, physical activity, consumption of vegetables etc., antihypertensive medication, blood sugar levels, body mass index	FINDRISK and similar scores	[35]
Physical inactivity	Time and intensity	[36]
Waist circumference	cm	[37]
Obesity	Body weight/body mass index	[38,39]
Visceral obesity	Fat mass/MRI	[40]
Liver fat	Fat mass/MRI	[41]
Insulin resistance	HOMA	[42]
Fasting hyperglycemia	glucose	[39]
Postprandial hyperglycemia	Oral glucose tolerance test, post meal glucose	[39]
HbA1c	HPLC	[39,43]
Waist circumference, blood pressure, mercury level, plasma triacylglycerol, blood glucose, HDL cholesterol, glucose	Support vector machines	[30]

**Table 2:** Basic descriptive statistics of sociodemographic, disease-related, and metabolomics parameters of the participants.

Parameter	Unit	n	Range Counts/Positive Responses	Mean ± SD
Demographics				
Age	Years	163	18–69	52.93 ± 12.7
Sex	-	163	6 men 101 women	-
BMI—body mass index	kg/m^2^	163	19.7–43.7	28.25 ± 5.04
Waist-hip ratio	-	163	0.6–1.1	0.92 ± 0.08
Systolic blood pressure	mm Hg	163	102–191	135.81 ± 16.91
Diastolic blood pressure	mm Hg	163	52–120	79.57 ± 11.99
**Prior or Concomitant Symptoms and Diseases**				
Head trauma	-	163	16 (9.8%)	-
Headache	-	163	37 (22.7%)	-
Postnasal drip	-	163	16 (9.8%)	-
Neurological disorder	-	163	6 (3.7%)	-
Renal dysfunction	-	163	6 (3.7%)	-
Nasal symptoms	-	163	0 = 101 (62%) 1 = 38 (23.3%) 2 = 15 (9.2%) 3 = 6 (3.7%) 4 = 2 (1.2%) 5 = 1 (0.6%)	-
Snoring	-	163	58 (35.6%)	-
Hepatitis	-	163	13 (8%)	-
Hypothyroidism	-	163	15 (9.2%)	-
Hyperthyroidism	-	163	8 (4.9%)	-
Surgery: palatine tonsils	-	163	20 (12.3%)	-
Surgery: pharyngeal tonsils	-	163	11 (6.7%)	-
Surgery: middle ear	-	163	4 (2.5%)	-
Surgery: teeth	-	163	25 (15.3%)	-
Surgery: nasal sinuses	-	163	4 (2.5%)	-
Surgery: nasal septum	-	163	8 (4.9%)	-
Surgery: nasal turbinates	-	163	1 (0.6%)	-
Frequent nasal infections	-	163	20 (12.3%)	-
Nasal polyposis	-	163	8 (4.9%)	-
Nasal obstruction	-	163	13 (8%)	-
Increased nasal secretion	-	163	12 (7.4%)	-
Chronic sinusitis	-	163	15 (9.2%)	-
Allergic rhinitis	-	163	23 (14.1%)	-
**Exposure to Toxic Substances**				
Alcohol use	-	163	0 = 23 (14.1%) 1 = 122 (74.8%) 2 = 18 (11%)	-
Smoking behavior	-	163	0 = 103 (63.2%) 1 = 37 (22.7%) 2 = 23 (14.1%)	-
Professional exposure to chemicals	-	163	23 (14.1%)	-
**Metabolomics Data**				
Triglycerides in serum	mmol/L	163	0.49–7.9	1.57 ± 1.08
Total cholesterol	mmol/L	163	2.8–8.85	5.53 ± 1
LDL—low density lipoprotein	mmol/L	163	0.87–6.46	3.33 ± 0.83
HDL—high density lipoprotein	mmol/L	163	0.67–2.84	1.51 ± 0.43
Hba1c—glycated hemoglobin	mmol/L	163	4.7–6.8	5.6 ± 0.4
Glucose, baseline	mmol/L	163	3.88–7.45	5.33 ± 0.69
Glucose, after 120 min	mmol/L	162	2.65–16.48	6.8 ± 2.31
Glucose, change	mmol/L	162	−3.68–9.56	1.46 ± 1.99
Fatty acids, baseline	mmol/L	163	0.12–93	1.03 ± 7.25
Fatty acids, after 120 min	mmol/L	162	0.01–0.25	0.06 ± 0.04
Fatty acids change	mmol/L	162	−92.97–0.07	−0.97 ± 7.28
Proinsulin, baseline	mmol/L	163	0.6–57.2	11.31 ± 10.72
Proinsulin, after 120 min	pmol/L	162	0.6–261	46.02 ± 50.07
Proinsulin, change	pmol/L	162	−0.3–213.7	34.66 ± 43.79
C-Peptide, baseline	pmol/L	163	296–3510	878.02 ± 390.38
C-Peptide after 120 min	pmol/L	162	500–8165	3034.72 ± 1365.23
C-Peptide, change	pmol/L	162	−318–6987	2155.36 ± 1180.24
Insulin, baseline	pmol/L	163	9–1157	79.24 ± 105.04
Insulin, after 120 min	pmol/L	161	26–2455	500.27 ± 452.14
Insulin, change	pmol/L	161	−293–2271	420.97 ± 421.16

**Table 3:** Classification performance measures for correctly assigning subjects to two diabetes risk classes, either lower (FINDRISK scores ≤ 11) or higher (FINDRISK score > 11) risk, obtained when training five different classifiers (random forests (RF), boosted classification and regression trees (ADA), C5.0 non-hierarchical decision rules, logistic regression (regression), and support vector machines (SVM)) with the metabolomics or olfactory information. The results represent the medians and 95% confidence intervals of the performance measures obtained during 1000 runs using class-proportional random divisions of the data set into disjoint training (two-thirds of the data set) and testing (one third) data subsets. The classifiers were trained on the metabolomics information, comprising d = 15 markers, on the olfactory information, comprising d = 3 olfactory subtest results, and on both types of information. With the metabolomics information for which the classifiers were successfully trained, training was repeated with randomly permuted data, which served as a negative control to detect possible overfitting. Results obtained when training was performed with permuted data are only shown when the training with original data had been successful.

Parameter	Classifier Performance									
**Data**	**Metabolomics Data Only**									
	Original					Permuted				
**Algorithm**	RF	ADA	C5.0	Regression	SVM	RF	ADA	C5.0	Regression	SVM
**Sensitivity, recall**	69 (48.3–86.2)	65.5 (44.8–82.8)	65.5 (37.9–86.3)	72.4 (55.2–89.7)	72.4 (55.2–86.2)	58.6 (34.5–79.3)	51.7 (31–72.4)	75.9 (13.8–100)	55.2 (34.4–79.3)	58.6 (34.5–82.8)
**Specificity**	64 (44–80)	60 (40–80)	64 (28–92)	64 (44–84)	64 (44–84)	40 (16–64)	48 (24–72)	24 (0–88)	42 (16–68)	40 (16–72)
**Positive predictive value, precision**	67.7 (57.6–79.2)	65.8 (53.8–80)	66.7 (53.8–83.3)	70 (59.4–82.8)	70 (59–83.3)	53.1 (40–68.2)	53.6 (37.9–69)	53.7 (33.3–73.3)	53.3 (36–71.1)	53.1 (36.4–70.8)
**Negative predictive value**	62.5 (50–76.9)	60 (46.4–75)	60 (46.7–76.2)	66.7 (54.2–81.3)	65.5 (53.6–80)	45.5 (25–68)	46.2 (28–63.6)	45.8 (9.5–93.5)	45.8 (21.7–69.6)	45.5 (22.2–69.2)
**F1**	67.8 (54.9–78)	65.5 (52–77.2)	65.5 (47.3–76.9)	71.4 (60–81.4)	70.4 (59.3–80)	56.7 (38.1–71.4)	53.6 (35.3–68.9)	64.1 (19–72)	55.2 (35.1–72.7)	55.6 (35.1–72.1)
**Balanced Accuracy**	65 (54.4–75.7)	63 (50.1–74.5)	63.3 (50.2–73.9)	68.2 (56.8–79.4)	67.9 (56.5–77.9)	49.3 (34.1–66.2)	49.9 (33.2–66.5)	50 (34.1–64.1)	49.6 (30.7–68.8)	49.3 (30.7–69)
**ROC-AUC**	73.3 (62.1–83.2)	68.8 (53.8–81.7)	65.2 (51.7–76.7)	76.4 (64.4–86.8)	67.9 (56.5–77.9)	49.2 (28.3–71)	55.6 (45.5–71.7)	50 (33.5–65.1)	60.1 (47–76.8)	55.3 (37.9–71.1)
**Data**	**Metabolomics and Olfactory Data**					**Olfactory Data Only**				
	Original					Original				
**Sensitivity, recall**	69 (48.3–82.8)	65.5 (44.8–79.3)	65.5 (37.9–86.2)	69 (51.7–86.2)	69 (51.7–86.2)	58.6 (41.4–75.9)	55.2 (37.9–72.4)	100 (17.2–100)	69 (44.8–93.1)	82.8 (41.4–100)
**Specificity**	64 (44–80)	64 (40–80)	60 (36–88)	64 (44–84)	64 (44–84)	44 (24–64)	48 (28–68)	0 (0–76)	28 (8–48)	16 (0–48)
**Positive predictive value, precision**	68 (58.1–80)	66.7 (54.3–78.6)	65.7 (53.8–81.5)	69 (59–82.1)	69 (58.1–81.8)	55.6 (45.8–65.4)	55.9 (45–66.7)	53.7 (41.7–59.1)	52.6 (44.1–58.5)	53.5 (44.8–56.3)
**Negative predictive value**	62.5 (51.5–76.9)	60 (47.4–73.9)	60 (46.4–73.9)	64.3 (52–78.9)	64 (52.2–78.9)	50 (35.3–61.5)	48.3 (35–61.5)	45.8 (30.8–50)	43.8 (25–65)	41.7 (16–62.5)
**F1**	67.8 (54.9–78.1)	65.5 (51.7–76.7)	65.4 (47.6–75.9)	69.1 (57.1–80)	69 (56.6–79.3)	57.1 (43.6–67.7)	55.7 (42.3–66.7)	69.9 (24.4–69.9)	59.7 (45.2–69.9)	64.9 (44–69.9)
**Balanced Accuracy**	65 (54.8–75.1)	63 (50.8–74.5)	62.8 (50.1–73.7)	66.8 (55.6–77.7)	66.5 (54.8–76.8)	52.2 (41.3–62.5)	52.1 (40.4–63.6)	50 (41.6–54.4)	48.5 (37.6–57.1)	49.7 (38.8–53.9)
**ROC-AUC**	74 (62.8–83.7)	68.8 (55–81.5)	65 (52.1–76.1)	74.5 (63.3–85.5)	66.5 (54.8–76.8)	55 (43.3–65.5)	54.8 (45.4–65.2)	50 (41.5–54.4)	54.2 (45.2–64.3)	50 (40.5–57.3)

**Table 4:** Classification performance measures for correctly assigning subjects to olfactory dysfunction categories, either reduced anosmia function (*n* = 1) or hyposmia, or normal olfactory function, obtained when training five different classifiers (random forests (RF), boosted classification and regression trees (ADA), C5.0 non-hierarchical decision rules, logistic regression (regression), and support vector machines (SVM)) with the metabolomics or olfactory information. The results represent the medians and 95% confidence intervals of the performance measures obtained during 1000 runs using class-proportional random divisions of the data set into disjoint training (two-thirds of the data set) and testing (one-third) data subsets. The classifiers were trained on the metabolomics information, comprising d = 15 markers, on the olfactory information, comprising d = 3 olfactory subtest results, and on both types of information. With the olfactory information for which the classifiers were successfully trained, training was repeated with randomly permuted data, which served as a negative control to detect possible overfitting. Results obtained when training was performed with permuted data are only shown when the training with original data had been successful.

Parameter	Classifier Performance									
**Data**	**Metabolomics Data Only**					**Metabolomics and Olfactory Data**				
	Original					Original				
**Algorithm**	RF	ADA	C5.0	Regression	SVM	RF	ADA	C5.0	Regression	SVM
**Sensitivity, recall**	0 (0–7.7)	23.1 (0–46.2)	0 (0–31)	0 (0–15.4)	0 (0–0)	76.9 (53.8–100)	76.9 (46.2–92.3)	76.9 (46.2–100)	84.6 (53.8–100)	84.6 (53.8–100)
**Specificity**	97.6 (90.2–100)	73.2 (56.1–85.4)	100 (65.9–100)	90.2 (75.6–100)	100 (100–100)	95.1 (87.8–100)	92.7 (82.9–100)	92.7 (80.5–100)	92.7 (80.5–100)	95.1 (87.8–100)
**Positive predictive value, precision**	0 (0–100)	20 (0–38.5)	20 (0–40.1)	8.3 (0–50)	0 (0–0)	84.6 (69.2–100)	78.6 (57.1–100)	76.9 (55.6–100)	78.6 (57.1–100)	85.7 (66.7–100)
**Negative predictive value**	75.9 (74.5–77.4)	74.4 (68.4–80.5)	75.9 (72.1–78)	75 (71.4–77.8)	75.9 (75.9–75.9)	93 (86.9–100)	92.7 (85.4–97.6)	92.5 (84.1–100)	94.9 (86.7–100)	95 (86.7–100)
**F1**	13.3 (11.1–14.3)	21.4 (7.4–38.7)	19 (8.3–37.4)	11.8 (8.3–25)	#WERT!	81.5 (63.6–92.9)	76.6 (58.3–88.9)	76 (52.6–88.9)	80 (60.8–92.9)	83.3 (63.6–96.3)
**Balanced Accuracy**	50 (46.3–53.8)	46.9 (35.4–58.6)	50 (41.7–54.4)	47.6 (40.2–54.2)	50 (50–50)	87.2 (74.7–96.3)	84.3 (71.9–93.7)	83.6 (68.4–93.7)	87.4 (73.3–97.6)	88.6 (74.5–98.8)
**ROC-AUC**	42.1 (28.1–55.9)	57 (45.6–72.1)	50 (40.2–53.4)	57.6 (45.2–71.1)	50 (50–50)	97 (91.7–99.8)	94.4 (83.3–98.9)	85.5 (70.6–95.1)	90.9 (74.9–98.9)	88.6 (74.5–98.8)
**Data**	**Olfactory Data Only**									
	Original					Permuted				
**Sensitivity, recall**	84.6 (53.8–100)	76.9 (46.2–100)	76.9 (46.2–100)	92.3 (69.2–100)	92.3 (61.5–100)	7.7 (0–38.5)	23.1 (0–61.5)	0 (0–30.8)	0 (0–15.6)	0 (0–0)
**Specificity**	97.6 (90.2–100)	95.1 (85.4–100)	95.1 (82.9–100)	97.6 (92.6–100)	97.6 (92.7–100)	92.7 (78–100)	73.2 (56.1–87.8)	100 (87.7–100)	100 (95.1–100)	100 (100–100)
**Positive predictive value, precision**	90 (70.6–100)	81.8 (63.6–100)	80 (58.8–100)	91.7 (75–100)	92.3 (78.6–100)	22.2 (0–100)	25 (0–50)	50 (0–100)	50 (0–100)	#WERT!
**Negative predictive value**	95.1 (87–100)	92.9 (85.4–100)	92.9 (84.8–100)	97.5 (90.5–100)	97.4 (89.1–100)	75.9 (71.7–82.5)	76.2 (67.6–85.7)	75.9 (74.5–80.9)	75.9 (75.5–78.9)	75.9 (75.9–75.9)
**F1**	84.6 (66.7–96.3)	78.4 (60–92.3)	78.6 (55.6–92.3)	88.9 (74.1–100)	91.7 (76.2–100)	20 (9.1–50)	26.1 (7.1–51.9)	36.4 (8.3–80)	14.3 (13.2–63.2)	#WERT!
**Balanced Accuracy**	89.9 (75.7–98.8)	85 (72–95.1)	86 (69.8–96.2)	93.7 (81–100)	92.5 (80.8–100)	50 (40.2–65.4)	50.6 (34.1–68.6)	50 (46.3–61.8)	50 (48.8–57.7)	50 (50–50)
**ROC-AUC**	97.8 (93.4–100)	96.1 (86.1–99.4)	87 (70.6–96.2)	98.4 (94.6–100)	92.5 (80.8–100)	54.2 (20.3–83.1)	58.5 (44.5–76.5)	50 (46.3–64.2)	81 (48.4–99.4)	50 (50–50)

## Data Availability

Data available on request from the last author.
